# P-498. One Small Step for Man, One Giant Leap for Ending the HIV Epidemic

**DOI:** 10.1093/ofid/ofae631.697

**Published:** 2025-01-29

**Authors:** Yasmeen Mann, Thomas Hagerman, Allison Malick, Sanjana Rao, Charlalynn Harris, Maria Santana-Garcés, Jacob Manteuffel, Smitha Gudipati, Indira Brar

**Affiliations:** Henry Ford Hospital, Detroit, Michigan; Henry Ford Hospital, Detroit, Michigan; Henry Ford Hospital, Detroit, Michigan; Wayne State University School of Medicine, Detroit, Michigan; Henry Ford Health System, Duluth, Georgia; Henry Ford Health, Livonia, Michigan; Henry Ford Hospital, Detroit, Michigan; Henry Ford Health System, Duluth, Georgia; Henry Ford Hospital, Detroit, Michigan

## Abstract

**Background:**

To achieve the goal to “End the HIV Epidemic” by 2030, unique interventions are needed to increase testing and subsequently link people with HIV (PWH) to care for rapid start of antiretroviral therapy (ART). Extending testing to include emergency department (ED) based HIV screening initiatives are effective in new case identification, earlier detection, and are encouraged by Centers for Disease Control and Prevention guidelines. Collaborations of infectious diseases (ID) providers with ED providers provides improved linkage to care for HIV and rapid initiation of ART. We describe our combined ED and ID HIV testing and linkage to care program.

**Figure:**
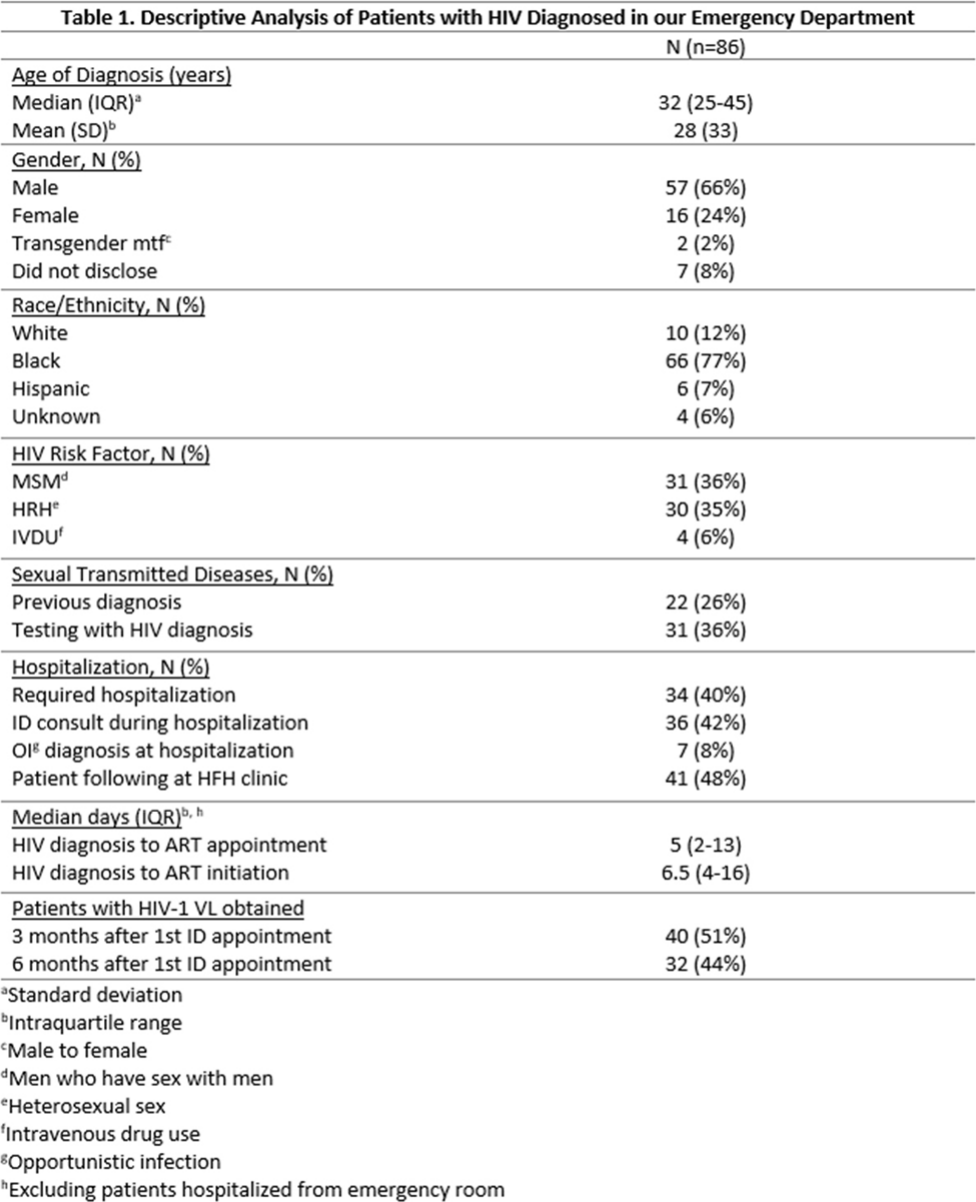

**Methods:**

This is a retrospective analysis of newly diagnosed PWH at Henry Ford Health (HFH) via reactive HIV fourth generation test by an ED based opt-out HIV screening program from 7/16/2020 – 10/28/2023. A best practice alert (BPA) prompted providers to order an HIV screening test for patients ages 18-65 years old without a previously documented HIV fourth generation test. Patients were informed of the test when ordered and could opt-out of testing if desired. Follow up and linkage to care was provided by a team of ID providers.

**Results:**

During the study period, a total of 48,725 fourth generation HIV screening tests were performed, of which 629 tests were reactive (1.24% of tests performed). Of the reactive tests, 86 patients (0.18% of all tests) were found to have a new diagnosis of HIV. Median CD4 cell count was 297 cells/mm3 (IQR: 98-614), 34 patients required hospitalization with 7 (8%) admitted with an opportunistic infection. Excluding the 34 patients hospitalized, the median time from positive result to first attended appointment was 5 days (IQR 2-13) and median time from screening test result to initiation of ART was 6.5 days (IQR: 4-16). At 3- or 6- months following HIV diagnosis, 40 (47%) had an HIV-1 follow-up viral load reported (see Table 1).

**Conclusion:**

HIV testing and early linkage to care are two key pillars of the “End the HIV Epidemic” initiative. As shown by our study, collaboration between ED and ID providers ensures increased testing in the ED, improved linkage to care, and rapid start of ART which will help in achieving the goals of these two pillars.

**Disclosures:**

**Indira Brar, MD**, Gilead: Advisor/Consultant|Gilead: Grant/Research Support|Gilead: Honoraria|Merck: Grant/Research Support|ViiV Healthcare: Grant/Research Support|ViiV Healthcare: Honoraria

